# Establishing Consensus on the Appropriate Tool for Measuring Adherence to Glaucoma Medication in a Sub‐Saharan African Population: A Multidisciplinary Delphi–Based Study

**DOI:** 10.1002/puh2.70222

**Published:** 2026-04-08

**Authors:** Benjamin Abaidoo, Khathutshelo Percy Mashige, Pirindhavellie Govender‐Poonsamy

**Affiliations:** ^1^ Ophthalmology Unit, Department of Surgery University of Ghana Medical School Accra Ghana; ^2^ Eye Department Korle Bu Teaching Hospital Accra Ghana; ^3^ Discipline of Optometry, School of Health Sciences University of KwaZulu‐Natal Durban South Africa

**Keywords:** Delphi technique, glaucoma, measurement, medication, non‐adherence

## Abstract

**Background:**

Despite the availability of various methods for assessing medication adherence, limited guidance exists regarding the most appropriate tool, particularly for measuring glaucoma medication adherence.

**Objective:**

To achieve expert consensus on the appropriate tool for measuring glaucoma medication adherence using the Delphi technique.

**Methods:**

A two‐round Delphi study was conducted with a panel of experts from diverse fields, assessing three validated adherence measurement tools. Consensus was determined using Kendall's Coefficient of Concordance. The extent of agreement and inter‐rater reliability were evaluated using the scale‐level content validity index (SCVI) and intraclass correlation coefficients (ICC), analysed in SPSS version 25.

**Results:**

Sixteen experts (mean age 53.8 ± 7.1 years; mean professional experience: 21.9 ± 6.8 years) participated. Consensus levels of 81.0% and 89.0% were achieved in the first and second rounds, respectively. Agreement on non‐adherence characteristics was high (SCVI and ICC values > 0.75). The most appropriate tool for measuring non‐adherence to glaucoma medication was the Glaucoma Treatment Compliance Assessment Tool‐Short form (GTCAT‐S) with an SCVI of 0.91 and ICC of 0.94 (95% CI: 0.78–0.99; *p* = 0.001).

**Conclusions:**

The GTCAT‐S was identified as the most suitable tool for measuring non‐adherence to glaucoma medication. It demonstrated a high SCVI and excellent inter‐rater reliability, indicating strong consensus among experts and robust measurement consistency.

## Graphical abstract

Expert consensus is achieved on the most appropriate tool for measuring glaucoma medication adherence using the Delphi technique. Sixteen specialists evaluate validated tools, and the Glaucoma Treatment Compliance Assessment Tool‐Short form (GTCAT‐S) emerges as the preferred measure, demonstrating high content validity and excellent inter‐rater reliability, ensuring robust assessment of non‐adherence in glaucoma care.

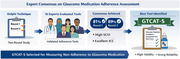

## 1 Introduction

Adherence to anti‐glaucoma medications has a significant impact on disease management [1, 2]. Measuring adherence to medication is a complex phenomenon that involves both objective and subjective techniques [3, 4, 5]. Several tools for measuring non‐adherence to glaucoma medication exist but have inherent limitations in addressing different aspects of non‐adherence [3, 4, 5]. Medication adherence in general is influenced by numerous behavioural factors exhibited by patients [4, 6, 7]. The European Society of Patient Adherence, Compliance and Persistence (ESPACOMP) group divides adherence into three interrelated phases, namely, initiation of medication, implementation and persistence with the medication prescribed [8]. These stages are also influenced by several behavioural factors, such as the patient's attributes, the environment of the patient, nature of the disease and its treatment [4, 8, 9]. For this reason, the choice of tools for measuring non‐adherence is very crucial as the development of effective and innovative interventions for addressing non‐adherence to medication depends on the quality of medication adherence studies [8].

Currently, there is no gold standard method for measuring non‐adherence to glaucoma medication [10]. According to Farmer [10] and Vermeire et al. [11], an ideal tool for measuring non‐adherence should be cost‐effective, user‐friendly, feasible, highly reliable and flexible in a busy clinic. Considering the multifactorial nature of non‐adherence, it is important to have an appropriate tool for measuring non‐adherence to glaucoma medication, as this could also be used to assess the effectiveness of interventions aimed at improving medication adherence.

The Delphi technique harnesses experts’ opinions and is useful in resolving situations where there are several multifaceted and interrelated factors with uncertainties [12, 13, 14]. Thus, the Delphi technique can be used for consensus‐building in situations where literature evidence about the most appropriate tool for measuring adherence is lacking [12]. This technique enables the use of qualitative and quantitative measures to strengthen the in‐depth understanding of experts’ opinions and provides a foundation for statistically analysing experts’ consensus [12, 13, 14].

The use of the Delphi technique is yet to be explored in selecting appropriate tools for assessing non‐adherence behaviours in patients with glaucoma. In this Delphi study, a multidisciplinary and patient‐centred approach was used to bring together patient groups, eye care providers, health policy planners and experts from other relevant fields in order to elicit consensus on the appropriate tool for measuring non‐adherence to glaucoma medications in sub‐Saharan Africa.

## 2 Methods

### 2.1 Study Design

This study used a two‐round Delphi iterative consultation design with a panel of experts from various fields to establish consensus on the appropriate tool for measuring non‐adherence to glaucoma medication. The principal investigator facilitated the Delphi process.

### 2.2 Inclusion Criteria

The participants were purposefully selected to ensure representation from patients, eye care providers and other key stakeholders in eye care and research. This diversity of perspectives was intended to enrich the decision‑making process by incorporating lived patient experiences, clinical expertise and broader system‑level insights. Participants were experts in ophthalmology, pharmacy, health economics, biostatistics, public health and a representative from the Glaucoma Association of Ghana (a non‐governmental organisation for patients with glaucoma in Ghana). Participants had to have at least 10 years of experience in their expert fields, a good professional image and be willing to be part of the process through all the stages and from diverse geographical locations (Africa, Europe and North America).

### 2.3 Exclusion Criteria

Experts from fields other than the fields described in the inclusion criteria, those with less than 10 years’ experience in their field of expertise and those unwilling to be part of all the stages of the study, were excluded.

### 2.4 Sample Size and Sampling

The sample size was determined on the basis of the study by Cejudo and Carmen [15], who recommended a minimum of seven participants for a Delphi process. For this study, a panel of 16 experts in the fields described above were included in the process to increase participation. Purposive and snowball sampling techniques were used to select participants. This was done by purposively selecting participants who met the inclusion criteria from the fields of expertise described above after they were approached and the purpose of the study **was** explained to them. Others were also recruited on the basis of recommendations from invited participants (snowball sampling). All participants expressed their interest to participate by consenting.

### 2.5 Response Rate

A minimum response rate of 70% in each round was required to reduce response bias [16]. To increase the response rate in this study, the principal investigator created a cordial rapport with participants through e‐mails and telephone communication.

### 2.6 Analysis of Experts’ Competence Level

The experts’ competence level in this Delphi process was analysed using the *K* coefficient, an index used in a previous study [16]. This factor was based on experts’ level of knowledge about non‐adherence to glaucoma medication using the knowledge assessment domain of the Glaucoma Treatment and Compliance Assessment Tool‐Short (GTCAT‐S) which included statements such as ‘my personal knowledge of the symptoms of glaucoma is excellent’, ‘a person can have glaucoma and not know it’, ‘eye pain is a common symptom of glaucoma’, ‘glaucoma treatments can prevent future vision loss’, and ‘vision lost from glaucoma is permanent’. Responses to these statements were reported in a 5‐point Likert scale from ‘disagree a lot—(1)’ to ‘agree a lot—(5)’. Scores were then computed and converted into percentages. Scores higher than or equal to 80% were classified as competent.

### 2.7 Definition of Consensus

To maintain rigour, Kendall's *W*, a non‐parametric test which measures the level of agreement for an expert panel, was used as the level of agreement was not normally distributed [16]. For Kendall's *W*, the level of consensus is considered strong where *W* ≥ 0.7 (70% or more), moderate 0.50–0.69 (50%–69%) and weak where *W* < 0.5 (less than 50%).

### 2.8 Data Collection Methods and Tools

This Delphi process had three stages, namely, the preliminary stage, the exploratory stage and the final stage. A coordinating group comprising the principal investigator (B.A.) and two other co‐authors (K.P.M. and P.G.P.) was formed for the preliminary stage. This group selected the top three self‐reported glaucoma disease‐specific medication adherence measurement tools through a systematic review of glaucoma disease‐specific methods for measuring adherence to glaucoma medications [17]. The three tools selected were the Glaucoma Treatment Compliance Assessment Tool‐Short form (GTCAT‐S) [18], the Eye‐Drop Satisfaction Questionnaire (EDSQ) [19] and the revised Glaucoma Adherence Questionnaire (GAQ‐R) [20].

At the exploratory stage, the selected tools were sent to the panel of experts via e‐mail in order to examine them according to the extent of agreement with adherence characteristics. As patient experiences are integral in assessing the reliability of patient‐reported outcome measures in health care [21, 22, 23], the conceptual foundation of this Delphi technique was supported by an exploratory qualitative study among 24 patients with glaucoma to identify barriers and motivators of adherence to glaucoma medication in a tertiary health facility. Details of this qualitative study are reported elsewhere [24]. From the qualitative study, knowledge about glaucoma, self‐efficacy, forgetfulness, missing doses, improper administration of medication, discontinuing medication, barriers to adherence, fear of blindness, perceived benefits from treatment and a good provider–patient relationship were identified as barriers and motivators of adherence. These factors were therefore used in assessing the extent of agreement with adherence characteristics to the three adherence tools.

The first‐round Delphi questionnaire was designed to determine which of the three self‐reported adherence tools best meets the factors derived from the qualitative study on a Likert scale (1–3: not at all appropriate, somewhat appropriate and very appropriate).

The experts were provided with text fields for comments at the end of each question. Responses from the first round were analysed, and comments were documented. Feedback was subsequently sent to the experts. In sequential rounds, the experts reviewed each tool and evaluated the degree of suitability of the items in the tool.

**FIGURE 1:**
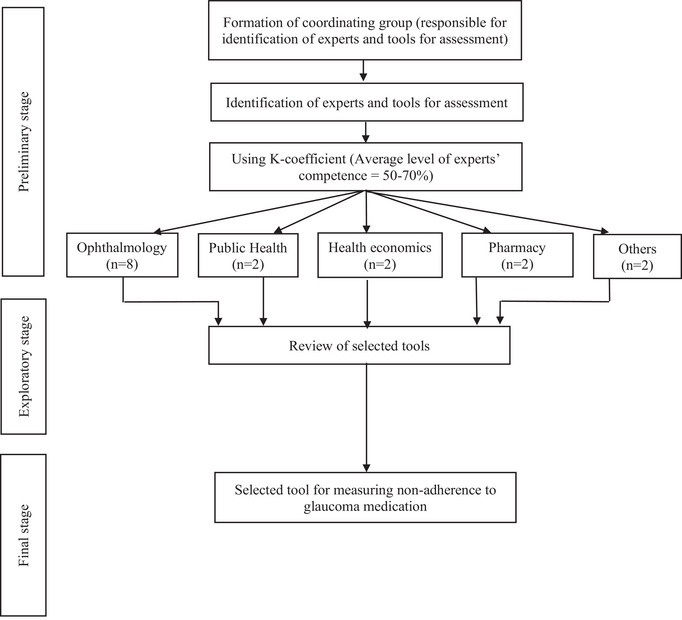
Delphi iterative consultation phase for the study. Others refer to the two representatives from the Glaucoma Association of Ghana.

During the final stage, the second‐round Delphi process, the most appropriate tool was selected after establishing consensus. The experts were asked to rate on a Likert scale (1–3: not at all appropriate, somewhat appropriate and very appropriate) which tool was most suitable for measuring non‐adherence to glaucoma medication in the context of the following factors: reliability and validity, relevance among patients with glaucoma, relevance within an African cultural/population setting, feasibility of implementation in a clinical setting and cost‐effectiveness. Text fields were provided at the end of each question for comments to be written. In each round, the *K* coefficient (the level of consensus) was calculated from the responses provided. A summary of the Delphi iterative consultation is illustrated in Figure 1.

### 2.9 Data Analysis

Descriptive statistics were used to describe the background characteristics of the Delphi experts. Competency levels were analysed using the *K* coefficient in accordance with the level of knowledge on adherence to glaucoma medication among the participants. The extent of agreement with non‐adherence behaviours and the most appropriate tool for measuring non‐adherence to glaucoma medication were examined using the scale level content validity index (SCVI) and intraclass correlation coefficients (ICC) [25]. These indices were calculated on the basis of the scores from the Likert scale ratings, where the critical cut‐off point for an agreement was set at two and above (somewhat appropriate and very appropriate) [13]. The S‐CVI which measures the content validity of the overall scale was calculated by averaging all the item‐level content validity indices (I‐CVI). The ICC which is a measure of the reliability of the experts’ ratings was computed using reliability analysis with SPSS version 25 (SPSS Inc, Chicago, IL) following a two‐way random effect model with multiple raters and an absolute agreement. ICC values less than 0.5 represented poor reliability, 0.5–0.75 represented moderate reliability, 0.75–0.9 represented good reliability, and values > 0.90 represented excellent reliability [16]. Open‐ended responses provided by Delphi panellists were analysed using qualitative content analysis. Two independent researchers [B.A., K.P.M.] conducted the analysis, beginning with familiarisation and initial coding of the data. Codes were then grouped into broader themes through iterative discussions. To ensure analytical rigour, discrepancies in coding or theme interpretation were resolved through consensus. When disagreements persisted, a third reviewer was consulted to reach a final decision. This structured approach enhanced the credibility and trustworthiness of the thematic insights derived from the panel's qualitative feedback. The level of significance was set at 5% with a 95% confidence interval.

### 2.10 Ethics Approval Statement

The study adhered to the ethical principles outlined in the Helsinki Declaration for medical research involving human subjects. Ethical approval was obtained from the Biomedical Research Ethics Committee of the University of KwaZulu‐Natal (BREC/00002965/2021) and the Institutional Review Board of a Teaching Hospital in Ghana (KBTH‐IRB/00048/2021).

## 3 Results

### 3.1 Characteristics of the Experts

Sixteen experts participated in the two‐round Delphi study, yielding a response rate of 80%. Their mean age was 53.8 ± 7.1 years (range = 41–66 years). Nine of the experts (56.3%) were males. The mean number of years’ experience was 21.9 ± 6.8 years (range = 10–40 years), and 12 participants were from Ghana. Other characteristics of the experts are shown in Table 1.

**TABLE 1:** Characteristics of the experts in the Delphi study.

Characteristics	Numbers	Percentages
Sex: Male Female Total	9 7 16	56.20 43.80 100.00
Area of expertise: Ophthalmology Public health Health economics/policy Pharmacy ^a^Others	8 2 2 2 2	50.00 12.50 12.50 12.50 12.50
Country of residence: Ghana India The United Kingdom The United States	12 1 2 1	75.00 6.25 12.50 6.25
Work setting: Academic Health	10 6	62.50 37.50

^a^ Other; representative from the Glaucoma Association of Ghana.

### 3.2 Competence Level (*K*) of the Experts

The value of *K* calculated was 82% (*n* = 16).

### 3.3 Level of Consensus at Each Stage

For this study, a Kendall's *W* ≥ 0.7 (70%) was achieved for each round of the Delphi process. Consensus levels of 81.0% and 89.0% were achieved in the first and second rounds of the survey, respectively.

### 3.4 Assessing the Extent of Agreement With Adherence Characteristics

In the first round of the Delphi process, the GTCAT‐S had the highest SCVI of 0.90 with an ICC of 0.85 (0.66–0.96; *p* = 0.001) (Table 2). The GTCAT‐S had an absolute agreement (CVI = 1) in seven domains, namely, knowledge about glaucoma, self‐efficacy, forgetfulness, missing doses, barriers to adherence, fear of blindness and a good provider–patient relationship (Table 2).

**TABLE 2:** Extent of agreement for 16 experts with 10 non‐adherence characteristics.

Tool	Characteristics	No. of Experts in agreement (ICVI)																No. of Ag.	ICV
		01	02	03	04	05	06	07	08	09	10	11	12	13	14	15	16		
GTCAT‐S	Knowledge about glaucoma	3	3	3	3	3	3	3	3	3	2	3	3	3	3	3	3	16	1.00
	Self‐efficacy	3	3	3	3	3	3	3	3	3	3	3	3	3	3	3	3	16	1.00
	Forgetfulness	3	3	3	3	3	3	3	3	3	3	3	3	3	3	3	3	16	1.00
	Missing doses	3	3	3	3	3	3	3	3	3	3	3	3	3	3	3	3	16	1.00
	Improper administration of medication	2	2	2	2	2	3	3	3	3	3	3	3	3	3	3	3	11	0.69
	Discontinuing medication	2	2	2	2	2	2	2	3	3	3	3	3	3	3	3	3	9	0.56
	Barriers to adherence	3	3	3	3	3	3	3	3	3	3	3	3	3	3	3	3	16	1.00
	Fear of blindness	3	3	3	3	3	3	3	3	3	3	3	3	3	3	3	3	16	1.00
	Perceived benefits from treatment	3	3	3	3	3	3	3	3	3	3	3	3	2	2	2	2	12	0.75
	A good provider–patient relationship	3	3	3	3	3	3	3	3	3	3	3	3	3	3	3	3	16	1.00
SCVI																			0.90
ICC = 0.85 (0.66–0.96; *p* = 0.001)																			
GAQ‐R	Knowledge about glaucoma	3	3	3	3	3	3	3	3	3	3	3	3	3	3	3	3	16	1.00
	Self‐efficacy	3	3	3	3	3	3	3	3	3	3	3	3	3	2	2	2	13	0.81
	Forgetfulness	2	3	3	3	3	3	3	3	3	3	2	2	2	2	2	2	9	0.56
	Missing doses	2	3	3	3	3	3	3	3	3	3	3	3	3	3	3	3	15	0.94
	Improper administration of medication	2	2	2	2	2	3	3	3	3	3	3	3	3	3	3	3	11	0.69
	Discontinuing medication	2	2	2	2	2	2	2	2	2	2	3	3	3	3	3	3	6	0.38
	Barriers to adherence	2	3	3	3	3	3	3	3	3	3	3	3	3	3	3	3	15	0.94
	Fear of blindness	3	3	3	3	3	3	3	3	3	3	3	3	3	3	3	3	16	1.00
	Perceived benefits from treatment	2	2	2	3	3	3	3	3	3	3	3	3	3	3	3	3	13	0.81
	A good provider–patient relationship	3	3	3	3	3	3	3	3	3	3	2	2	2	2	2	2	10	0.63
SCVI																			0.78
ICC = 0.79 (0.55–0.94; *p* = 0.001)																			
EDSQ	Knowledge about glaucoma	3	3	3	3	3	3	3	3	3	3	3	3	3	3	3	3	16	1.00
	Self‐efficacy	3	3	3	3	3	3	3	3	3	3	2	2	2	2	2	2	10	0.63
	Forgetfulness	3	3	3	3	3	3	3	3	3	3	3	3	3	3	3	3	16	1.00
	Missing doses	3	3	3	3	3	3	3	3	3	3	3	3	3	3	3	3	16	1.00
	Improper administration of medication	2	2	2	2	2	2	2	3	3	3	3	3	3	3	3	3	9	0.56
	Discontinuing medication	3	3	3	3	3	3	3	3	3	3	3	3	3	3	3	3	16	1.00
	Barriers to adherence	3	3	3	3	3	3	3	3	3	3	2	2	2	2	2	2	10	0.63
	Fear of blindness	2	2	2	2	2	2	2	3	3	3	3	3	3	3	3	3	9	0.56
	Perceived benefits from treatment	2	2	2	2	2	2	2	3	3	3	3	3	3	3	3	3	9	0.56
	A good provider–patient relationship	2	2	2	2	2	2	2	3	3	3	3	3	3	3	3	3	9	0.56
SCVI																			0.75
ICC = 0.82 (0.61–0.95; *p* = 0.001)																			

*Note:* Likert scale (1–3: not at all appropriate, somewhat appropriate and very appropriate). No. of Ag. = number of agreements.

Abbreviations: ICC, intraclass correlation coefficient; ICVI, item content validity index; SCVI, scale level content validity index.

Knowledge about glaucoma was the only domain with an absolute agreement (CVI = 1) among the experts for all the three adherence tools (Table 2).

From the first‐round survey, open‐ended responses were analysed qualitatively, and several recurring themes emerged. One prominent theme was the importance of patient knowledge and education, with panellists emphasising that understanding glaucoma significantly influences medication adherence. Other themes included perceived severity of disease, trust in healthcare providers and accessibility of medication.

### 3.5 The Most Appropriate Tool for Measuring Non‐Adherence to Glaucoma Medication

An absolute agreement (CVI = 1) was realised among the experts in the domain of reliability and validity, relevance among patients with glaucoma and feasibility of implementation in a clinical setting for the GTCAT‐S.

At the second round, in analysing the most appropriate tool for measuring non‐adherence to glaucoma medication adherence, there was a consensus in favour of the GTCAT‐S with an SCVI of 0.91 with ICC value of 0.94 (95% CI:0.78–0.99; *p* = 0.001) compared to the GAQ‐R [SCVI = 0.90; ICC = 0.88 (95% CI:0.57–0.99; *p* = 0.001)] and the EDSQ [SCVI = 0.86; ICC = 0.78 (95% CI:0.17–0.98; *p* = 0.016)] respectively (Table 3).

**TABLE 3:** The most appropriate tool for measuring non‐adherence to glaucoma medication.

Tool	Sub‐category	Number of experts in agreement (ICVI)																No. of Ag.	ICV
		01	02	03	04	05	06	07	08	09	10	11	12	13	14	15	16		
GTCAT‐S	Reliability and validity	3	3	3	3	3	3	3	3	3	3	3	3	3	3	3	3	16	1.00
	Relevance among patients with glaucoma	3	3	3	3	3	3	3	3	3	3	3	3	3	3	3	3	16	1.00
	Relevance within an African cultural/population setting	3	3	3	3	3	3	3	3	3	3	3	3	3	3	2	2	14	0.88
	Feasibility of implementation in a clinical setting	3	3	3	3	3	3	3	3	3	3	3	3	3	3	3	3	16	1.00
	Cost‐effectiveness	2	2	3	3	3	3	3	3	3	3	3	3	3	2	2	2	11	0.69
SCVI																			0.91
ICC = 0.94 (0.78–0.99; *p* = 0.001)																			
																			
GAQ‐R	Reliability and validity	3	3	3	3	3	3	3	3	3	3	3	3	3	3	3	3	16	1.00
	Relevance among patients with glaucoma	2	3	3	3	3	3	3	3	3	3	3	3	3	3	3	3	15	0.93
	Relevance within an African cultural/population setting	3	3	3	3	3	3	3	3	3	3	3	3	3	3	2	2	14	0.88
	Feasibility of implementation in a clinical setting	3	3	3	3	3	3	3	3	3	3	3	3	3	3	3	3	16	1.00
	Cost‐effectiveness	2	2	3	3	3	3	3	3	3	3	3	3	3	2	2	2	11	0.69
SCVI																			0.90
ICC = 0.88 (0.57–0.99; *p* = 0.001)																			
																			
EDSQ	Reliability and validity	3	3	3	3	2	2	3	3	3	3	3	3	3	3	3	3	14	0.88
	Relevance among patients with glaucoma	3	3	3	3	3	3	3	3	3	3	3	3	3	3	3	3	16	1.00
	Relevance within an African cultural/population setting	3	3	3	3	3	3	3	3	3	3	3	3	2	2	2	2	12	0.75
	Feasibility of implementation in a clinical setting	3	3	3	3	2	2	3	3	3	3	3	3	3	3	3	3	14	0.88
	Cost‐effectiveness	3	3	3	3	2	2	2	3	3	3	3	3	3	3	3	3	13	0.81
SCVI																			0.86
ICC = 0.78 (0.17–0.98; *p* = 0.016)																			

*Note:* Likert scale (1–3: not at all appropriate, somewhat appropriate and very appropriate). No. of Ag. = number of agreements.

Abbreviations: ICC, intraclass correlation coefficient; ICVI, item content validity index; SCVI, scale level content validity index.

From the second‐round survey comments, four participants mentioned that the issue of relevance within an African cultural/population setting was quite difficult to assess because Africa is a huge place with many different cultures, and in some settings, folks are happy to say what they think, and in others, the culture of politeness may preclude true response gathering.

## 4 Discussion

As the Delphi method is used extensively in consensus‐based solutions [13], it was applied in this study to determine the most appropriate tool for measuring medication adherence. The extent of agreement with non‐adherence characteristics in relation to the tools was high (SCVI and ICC values > 0.75) with 81.0% consensus. The high ICCs in the first round demonstrate that the experts agreed on the importance of the non‐adherence characteristics used in assessing the tools. Similar to our findings, a high content validity index with an excellent inter‐rater agreement was reported in a study to investigate the content validity of a new patient‐reported experience measure [26]. In addition, Almathami et al. [27] reported good inter‐rater agreement among Delphi participants in identifying factors that influence users’ motivation toward the use of a teleconsultation system.

Knowledge about glaucoma was the only domain with an absolute agreement (ICV = 1) among the experts for all three adherence tools, signifying the importance of assessing the level of knowledge in the measurement of adherence. A prominent theme from the qualitative analysis of open‐ended responses from the first‐round Delphi was the importance of patient knowledge and education, with panellists consistently emphasising that a deeper understanding of glaucoma motivates patients to adhere to their medication regimen. This aligns with existing literature highlighting the role of health literacy in chronic disease management [28, 29, 30, 31]. Additional themes included the perceived severity of disease, which influences patients’ sense of urgency and commitment to treatment; trust in healthcare providers, which affects willingness to follow prescribed regimens; and accessibility of medication, a practical barrier that can undermine adherence despite good intentions. These findings underscore the multifactorial nature of adherence behaviour, suggesting that effective interventions must address both informational and systemic factors. This suggests the need to consider a tool that can assess the level of knowledge about glaucoma in a quest for the choice of a suitable adherence measurement tool.

The high SCVI and ICC values of the GTCAT‐S demonstrate excellent validity, reliability and robustness in measuring adherence to glaucoma medication. These indicate strong agreement among experts that the tool's items are relevant and representative of the construct being measured (adherence to glaucoma medication). This suggests that the instrument has strong content validity and is well‐aligned with the theoretical and practical dimensions of adherence behaviour.

Furthermore, its excellent inter‐rater reliability reflects consistent evaluations across panellists, reinforcing the tool's clarity, interpretability and applicability. Together, these metrics suggest that the GTCAT‐S is not only conceptually sound but also practically reliable, making it a strong instrument for use in both clinical and research settings. Its psychometric strength enhances confidence in its ability to generate meaningful, reproducible data across diverse assessors.

The constitution of experts with high academic achievements and stakeholders in the management of glaucoma may account for this high inter‐rater reliability with less differences among the experts. The COSMIN (Consensus‐based Standards for the Selection of Health Status Measurement Instruments) study, through a four‐round Delphi survey by international experts made up of psychologists, epidemiologists, statisticians and clinicians, justified the use of the SCVI and the ICC as significant measurement properties in assessing the appropriateness of patient‐reported measurement outcomes and the proposal of novel health measurement scales [21, 22, 23].

Our results reinforce the need for consensus building in the choice of adherence measurement tools in healthcare delivery and research. The experts’ agreement for a choice of glaucoma disease‐specific adherence measurement tool with excellent reliability, which is sensitive in identifying significant changes in the eye health of persons with glaucoma, also reflects a pragmatic approach to using a tool that will work best in a natural glaucoma clinic setting. The GTCAT‐S in various studies has demonstrated acceptable test–retest reliability and internal consistency and wide usage in assessing adherence among glaucoma patients [3, 32]. Our findings also build on the evidence that the Delphi technique could offer a more insightful way of making decisions in eyecare practice.

Participants mentioned the relevance of the tools within an African cultural/population setting. They indicated that it was difficult to assess because Africa is a large continent with many different cultures, and in some settings, people are comfortable to express their thoughts, and in others, there is pragmatic politeness that preclude true response gathering. Future studies should therefore take a critical look at this factor before using it as an assessment criterion. Nevertheless, the experts agreed that the other four factors (reliability and validity, relevance among patients with glaucoma, feasibility of implementation in a clinical setting and cost‐effectiveness) were significant in assessing the choice of an appropriate tool for assessing adherence to glaucoma medication.

The GTCAT's relative strengths lie in its theoretical foundation, multidimensional structure and contextual relevance, having been developed using the Health Belief Model, which captures behavioural, cognitive and contextual factors influencing adherence [18]. Unlike the EDSQ [19], which focuses primarily on patient satisfaction and administration experience, or the GAQ‐R [20], which emphasises self‐reported behaviours and barriers, the GTCAT offers a more comprehensive and explanatory framework. Its ability to assess underlying motivations and beliefs makes it particularly valuable for designing targeted interventions and adapting to diverse cultural contexts. These attributes contributed to its higher ratings and strong consensus among Delphi panellists.

Although the GTCAT‐S demonstrated superiority in the sub‐Saharan African context from the Delphi panel evaluation, this superiority may reflect the assessment criteria used. Factors such as language nuances, health literacy and patient expectations likely influenced the Delphi panel ratings. However, broader validation would be appropriate to assess its applicability across diverse global populations. Therefore, although the tool shows promise, its applicability in other geographical settings requires further validation to ensure its reliability, sensitivity and relevance.

Although our Delphi findings guide the selection of non‐adherence measurement instruments, the recommendations for an ideal tool should always be tailored to the specific need to be addressed. This study provides a systematic framework for reviewing and comparing existing adherence measurement tools. Experts in non‐adherence measurement have suggested the need to use multiple measurement tools in assessing medication non‐adherence in a study [4]. However, this suggestion will work when multiple non‐adherence characteristics are the key outcome of interest. The choice of a self‐reported measurement tool is essential in not only measuring the level of adherence but also in assessing significant elements of non‐adherence, such as reasons for non‐adherence, drug affordability and manual dexterity, among others [13, 33].

## 5 Strengths and Limitations

This study adopted a multidisciplinary approach in constituting a heterogenous group of experts with an international composition of experienced eye care providers, health policy planners, patients’ groups and experts from other relevant fields. This promoted a high level of consensus after the first and second rounds to improve the validity of the results and reflected different views. Anonymity was maintained which prevented group domination and enhanced participation. The sample size was reasonable, and the response rate was high for a Delphi study.

Despite the above‐mentioned strengths, there were limitations that need to be acknowledged. For instance, more time was required to administer the two rounds of the Delphi process in order to consolidate the outputs. Although the study utilised barriers and motivators of non‐adherence derived from a qualitative study by persons with glaucoma, the selections of questions submitted to the experts were mostly controlled by the facilitator of the Delphi process. Even though a higher level of agreement was achieved on each domain of the framework for assessing the tools, there was a lack of absolute agreement with most of the non‐adherence characteristics. Lastly, the GTCAT‑S showed superiority in the sub‑Saharan African context following the Delphi panel evaluation; however, this outcome was a result of the assessment criteria used. Language nuances, health literacy and patient expectations likely influenced the panel judgement. Broader validation across diverse global populations is therefore required to establish the tool's reliability, sensitivity and relevance beyond the sub‑Saharan African setting.

## 6 Conclusions

The study identified the GTCAT‐S tool as an appropriate tool for measuring non‐adherence to glaucoma medication. Consensus was achieved with a high ICC, demonstrating excellent inter‐rater reliability in the process. This outcome will guide clinicians and researchers in choosing the most appropriate tool for measuring adherence. Future research may focus on using the GTCAT‐S tool to measure adherence in randomised controlled trials.

## Author Contributions

Benjamin Abaidoo was responsible for conceptualisation, methodology, formal analysis, data collection and writing of drafts. Khathutshelo Percy Mashige and Pirindhavellie Govender‐Poonsamy were responsible for conceptualisation, methodology, analysis, resources, review of drafts and administration.

## Funding

This was a self‐funded project by the principal investigator.

## Disclosure

The views and opinions expressed in this article are those of the authors and are the product of professional research. It does not necessarily reflect the official policy or position of any affiliated institution, funder, agency or that of the publisher. The authors are responsible for this article's results, findings and content.

## Conflicts of Interest

The authors declare no conflicts of interest.

## Journal‐Specific Sections

Public Health Challenges.

## Data Availability

The authors confirm that the data supporting the findings of this study are available upon reasonable request through the corresponding author.

## References

[1] A. C. Zaharia , O. M. Dumitrescu , M. Radu , and R. E. Rogoz , “Adherence to Therapy in Glaucoma Treatment—A Review,” Journal of Personalized Medicine 12, no. 4 (2022): 514. 35455630 10.3390/jpm12040514 PMC9032050

[2] L. Quaranta , A. Novella , M. Tettamanti , L. Pasina , R. N. Weinreb , and A. Nobili , “Adherence and Persistence to Medical Therapy in Glaucoma: An Overview,” Ophthalmology and Therapy 12, no. 5 (2023): 2227–2240, 10.1007/s40123-023-00730-z. 37311908 PMC10441906

[3] J. Cho , L. M. Niziol , P. P. Lee , et al., “Comparison of Medication Adherence Assessment Tools to Identify Glaucoma Medication Nonadherence,” Ophthalmology Glaucoma 5, no. 2 (2022): 137–145. 34358735 10.1016/j.ogla.2021.07.012 PMC8814049

[4] P. V. Burkhart and E. Sabaté , “Adherence to Long‐Term Therapies: Evidence for Action,” Journal of Nursing Scholarship 35, no. 3 (2003): 207. 14562485

[5] W. Y. Lam and P. Fresco , “Medication Adherence Measures: An Overview,” BioMed Research International 2015 (2015): 217047. 26539470 10.1155/2015/217047 PMC4619779

[6] S. M. Chang , I. C. Lu , Y. C. Chen , C. F. Hsuan , Y. J. Lin , and H. Y. Chuang , “Behavioral Factors Associated With Medication Nonadherence in Patients With Hypertension,” International Journal of Environmental Research and Public Health 18, no. 18 (2021): 9614. 34574540 10.3390/ijerph18189614 PMC8469687

[7] D. Bott , A. Subramanian , D. Edgar , J. G. Lawrenson , and P. Campbell , “Barriers and Enablers to Medication Adherence in Glaucoma: A Systematic Review of Modifiable Factors Using the Theoretical Domains Framework,” Ophthalmic & Physiological Optics 44, no. 1 (2024): 96–114. 37985237 10.1111/opo.13245

[8] G. S. De , L. L. Zullig , and J. Dunbar‐Jacob , et al., “Improving Medication Adherence Research Reporting: ESPACOMP Medication Adherence Reporting Guideline (EMERGE),”European Journal of Cardiovascular Nursing 18, no. 4 (2019): 258–259. 30739497 10.1177/1474515119830298 PMC6433491

[9] K. Singh , A. Singh , D. Jain , and V. Verma , “Factors Affecting Adherence to Glaucoma Medication: Patient Perspective From North India,” Indian Journal of Ophthalmology 72, no. 3 (2024): 391–396, 10.4103/IJO.IJO_806_23. 38099369 PMC11001247

[10] K. C. Farmer , “Methods for Measuring and Monitoring Medication Regimen Adherence in Clinical Trials and Clinical Practice,” Clinical Therapeutics 21, no. 6 (1999): 1074–1090. 10440628 10.1016/S0149-2918(99)80026-5

[11] E. Vermeire , H. Hearnshaw , P. Van Royen , and J. Denekens , “Patient Adherence to Treatment: Three Decades of Research. A Comprehensive Review,” Journal of Clinical Pharmacy and Therapeutics 26, no. 5 (2001): 331–342. 11679023 10.1046/j.1365-2710.2001.00363.x

[12] J. Steurer , “The Delphi Method: An Efficient Procedure to Generate Knowledge,” Skeletal Radiology 40, no. 8 (2011): 959–961. 21667147 10.1007/s00256-011-1145-z

[13] I. M. Kronish , C. T. Thorpe , and C. I. Voils , “Measuring the Multiple Domains of Medication Nonadherence: Findings From a Delphi Survey of Adherence Experts,” Translational Behavioral Medicine: Practice, Policy, Research 11, no. 1 (2021): 104–113. 10.1093/tbm/ibz133 PMC7877304 31580451

[14] O. Marten , F. Koerber , D. Bloom , et al., “A DELPHI Study on Aspects of Study Design to Overcome Knowledge Gaps on the Burden of Disease Caused by Serogroup B Invasive Meningococcal Disease,” Health and Quality of Life Outcomes [Electronic Resource] 17, no. 1 (2019): 1–9. 31118091 10.1186/s12955-019-1159-0 PMC6532178

[15] L. Cejudo and M. D. Carmen , “Assessing Personal Learning Environments (PLEs). An Expert Evaluation,” Journal of New Approaches in Educational Research 2, no. 1 (2013): 39–44.

[16] P. Legendre , “Species Associations: The Kendall Coefficient of Concordance Revisited,” Journal of Agricultural, Biological and Environmental Statistics 10, no. 2 (2005): 226–245.

[17] B. Abaidoo , K. P. Mashige , P. Govender‐Poonsamy , N. N. Tagoe , V. A. Essuman , and S. Y. Adam , “Glaucoma Disease‐Specific Adherence Measurement Tools Validated for Measuring Adherence to Glaucoma Medications: A Systematic Review,” Health Science Reports 8 (2025): e70427. 39931261 10.1002/hsr2.70427 PMC11808388

[18] G. T. Barker and S. L. Mansberger , “Psychometric Properties of the Reduced Version of the Glaucoma Treatment Compliance Assessment Tool (GTCAT),” Ophthalmic Epidemiology 26, no. 1 (2019): 55–62. 30204034 10.1080/09286586.2018.1516785

[19] J. P. Nordmann , P. Denis , M. Vigneux , E. Trudeau , and I. Guillemin , and G. Berdeaux , “Development of the Conceptual Framework for the Eye‐Drop Satisfaction Questionnaire (EDSQ©) in Glaucoma Using a Qualitative Study,” BMC Health Services Research [Electronic Resource] 7 (2007): 1–9. 17683594 10.1186/1472-6963-7-124 PMC1973077

[20] T. A. Gray , C. Fenerty , and R. Harper , et al., “Individualised Patient Care as an Adjunct to Standard Care for Promoting Adherence to Ocular Hypotensive Therapy: An Exploratory Randomised Controlled Trial,” Eye (London, England) 26, no. 3 (2012): 407–417. 22094303 10.1038/eye.2011.269 PMC3299012

[21] C. B. Terwee , C. A. C. Prinsen , A. Chiarotto , et al., “COSMIN Methodology for Evaluating the Content Validity of Patient‐Reported Outcome Measures: A Delphi Study,” Quality of Life Research 27, no. 5 (2018): 1159–1170. 29550964 10.1007/s11136-018-1829-0 PMC5891557

[22] L. B. Mokkink , C. B. Terwee , D. L. Patrick , et al., “The COSMIN Checklist for Assessing the Methodological Quality of Studies on Measurement Properties of Health Status Measurement Instruments: An International Delphi Study,” Quality of Life Research 19, no. 4 (2010): 539–549. 20169472 10.1007/s11136-010-9606-8 PMC2852520

[23] H. G. Tegegn , S. Wark , E. Tursan d'Espaignet , and M. J. Spark , “Measurement Properties of Patient‐Reported Outcome Measures for Medication Adherence in Cardiovascular Disease: A COSMIN Systematic Review,” Clinical Drug Investigation 42, no. 11 (2022): 879–908. 36180813 10.1007/s40261-022-01199-7 PMC9617955

[24] B. Abaidoo , K. P. Mashige , and P. Govender‐Poonsamy , “Development of a Patient‐Centred Toolkit for Improving Glaucoma Medication Adherence: A Motivational Interviewing Approach,” Health Sciences Investigations Journal 6, no. 2 (2024): 892–902.

[25] D. Liljequist , B. Elfving , and K. S. Roaldsen , “Intraclass Correlation—A Discussion and Demonstration of Basic Features,” PLoS ONE 14, no. 7 (2019): e0219854. 31329615 10.1371/journal.pone.0219854 PMC6645485

[26] C. Bull , J. Crilly , S. Latimer , and B. M. Gillespie , “Establishing the Content Validity of a New Emergency Department Patient‐Reported Experience Measure (ED PREM): A Delphi Study,” BMC Emergency Medicine 22, no. 1 (2022): 1–10. 35397490 10.1186/s12873-022-00617-5 PMC8994175

[27] H. K. Y. Almathami , K. T. Win , and E. Vlahu‐Gjorgievska , “Development and Validation of a New Tool to Identify Factors That Influence Users' Motivation Toward the Use of Teleconsultation Systems: A Modified Delphi Study,” International Journal of Medical Informatics 157 (2022): 104618. 34741893 10.1016/j.ijmedinf.2021.104618

[28] M. F. Cabezas , G. Nazar , A. V. Ranchor , and C. Annema , “Effect of Health Literacy Interventions on Self‐Management in Chronic Diseases: A Systematic Review,” Annals of Behavioral Medicine 59, no. 1 (2025): kaaf073, 10.1093/abm/kaaf073. 41176327 PMC12579557

[29] L. Tsichla , E. Patelarou , E. Detorakis , M. K. Tsilimbaris , A. E. Patelarou , and K. Giakoumidakis , “Enhancing Health Literacy and Self‐Management in Glaucoma Patients: Evidence From a Nurse‐Led Educational Intervention,” Healthcare (Basel) 13, no. 8 (2025): 861, 10.3390/healthcare13080861. 40281810 PMC12027466

[30] K. W. Muir and P. P. Lee , “Health Literacy and Success With Glaucoma Drop Administration,” Ophthalmology Glaucoma 4, no. 1 (2021): 49–56, 10.1016/j.ogla.2021.07.004. 34052458

[31] V. A. Essuman , N. N. Tagoe , A. Essuman , et al., “A Cross‐Sectional Study of Ocular Changes in Children and Adolescents With Diabetes Mellitus in Selected Health Facilities in Ghana,” International Journal of Environmental Research and Public Health 19, no. 9 (2022): 5295, 10.3390/ijerph19095295. 35564690 PMC9104046

[32] F. G. Sanchez , S. L. Mansberger , and P. A. Newman‐Casey , “Predicting Adherence With the Glaucoma Treatment Compliance Assessment Tool,” Physiology & Behavior 176, no. 3 (2017): 139–148. 32740508 10.1097/IJG.0000000000001616 PMC7657978

[33] N. Ratanawongsa , A. J. Karter , M. M. Parker , et al., “Communication and Medication Refill Adherence the Diabetes Study of Northern California,” JAMA Internal Medicine 173, no. 3 (2013): 210–218. 23277199 10.1001/jamainternmed.2013.1216 PMC3609434

